# Factors associated with self-rated health among immigrant workers in South Korea: Analyzing the results of the 2020 survey on immigrants' living conditions and labor force

**DOI:** 10.3389/fpubh.2022.933724

**Published:** 2022-09-23

**Authors:** Soo Jin Kang, Jinseub Hwang, Dohyang Kim, Bongjeong Kim

**Affiliations:** ^1^Department of Nursing, Daegu University, Daegu, South Korea; ^2^Department of Bigdata Science, Daegu University, Gyeongsan, South Korea; ^3^Department of Statistics, Daegu University, Gyeongsan, South Korea; ^4^Department of Nursing, Cheongju University, Cheongju, South Korea

**Keywords:** immigrant workers, self-rated health, gender, interaction effect, National survey, South Korea

## Abstract

Immigrants' health is an emerging public health issue worldwide. This study aimed to measure immigrant workers' self-rated health and identify the factors affecting it. Data were obtained from the nationwide 2020 Survey on Immigrants' Living Conditions and Labor Force in Korea. The data from 14,277 economically active immigrants who participated in the study were analyzed. Self-rated health was measured using one question and divided into dichotomized categories (good and poor). Multivariate logistic regression with a weighted sampling method was used to investigate associated factors, namely, individual, social, and living and working environment variables, and to evaluate the interaction effects with gender. Overall, 23.0% of the participants showed poor self-rated health. The odds ratios for poor self-rated health were high in participants who reported unmet healthcare needs (OR = 3.07, 95% confidence interval: 3.00–3.13) compared to those who reported other factors, followed by moderate job satisfaction (OR = 2.23, 95% confidence interval: 2.20–2.26) and unsatisfied residential environment satisfaction (OR = 1.80, 95% confidence interval: 1.74–1.86). Significant associations were found between self-rated health and most variables, including the interaction test (gender × residential environment satisfaction, education level, working hours, and length of residence). To enhance immigrants' health status, the Korean government must develop strategies to increase their access to healthcare services and minimize unmet healthcare needs. In addition, working conditions must be improved, specifically regarding long working hours and discrimination; furthermore, immigrants' living environments should be considered.

## Introduction

Immigrant health is increasingly recognized as an important public health issue due to increased global migration. Therefore, attention must be paid to the health problems associated with labor migration among international immigrants (1).

The proportion of immigrants to the total population in Korea has been increasing yearly, from 3.69% in 2015 to 4.87% in 2019; however, this proportion temporarily decreased to 3.93% in 2020 due to the impact of COVID-19 (2). Immigrants in Korea are generally classified into naturalized citizens and foreigners, namely, migrant workers, overseas Korean nationals, international students, and permanent residents (3). Most of the naturalized citizens comprise married immigrants—for example, there were 15,341 married immigrants in 2020—contributing to these notable changes in Korea (4). Regarding foreigners, most people were from other Asian countries, namely, China (43.6%), Vietnam (8.9%), and other Southeast Asian countries (2). The majority of these immigrants were allowed to reside in Korea to meet the labor demand following a worker shortage in Korea.

Self-rated health (SRH) is the most widely used proxy for actual health status in survey research studies (5), and poor SRH is known to be a strong predictor of subsequent health outcomes and mortality (6, 7). Many studies have shown that a decline in immigrants' SRH is associated with length of residence (8), perceived discrimination (9, 10), lack of social support from family or neighbors, barriers to healthcare access (11, 12), and relatively poor living and working conditions (13, 14).

Unlike the United States or Europe, where multicultural societies were formed earlier, Korea has long been an ethnically and culturally homogenous society; therefore, there is little research on immigrant health in Korea. Some Korean studies regarding immigrant health have focused on specific groups, namely, migrant workers (15–17) and immigrant Asian women (18, 19). However, immigrant health across diverse immigrant groups in Korea is not well studied.

Previous studies have reported gender differences in immigrant SRH; immigrant men have more health advantages than immigrant women (20–22). Furthermore, the length of stay has been found to affect the SRH of various immigrant populations, and gender differences are associated with the length of stay in Korea, as seen from immigrant statistical data. In the 2020 immigration statistics of Korea, of the 173,756 registered married immigrants, 79.5% were women, and of the 455,287 migrant workers, 78.8% were male (2). Female Korean workers receive low wages compared to their male counterparts, mainly due to their increased responsibilities following marriage and childbirth; female immigrants in Korea also face gender discrimination in terms of employment opportunities and wages (19, 23). Therefore, to understand the factors affecting immigrants' health, the characteristics of immigrant men and women in Korean society must be recognized.

This study aims to investigate the SRH of employed immigrants and explore the factors affecting their SRH. Furthermore, it extends the analysis with interaction terms: between gender and other factors (education level, length of stay, working hours, and residential environment factors). Understanding the associated factors may guide stakeholders and researchers in assisting immigrants to successfully adapt to their new residences.

## Methods

### Study design and data

This study follows a cross-sectional design. It employs data from the 2020 Survey on Immigrants' Living Conditions and Labor Force, conducted from May 18 to June 2, 2021, by the Ministry of Justice and Statistics, Korea. The survey aimed to identify the current living and employment conditions of foreigners aged 15 years or older (workers, overseas Korean nationals, and foreign students) and naturalized persons (married immigrants) living in Korea. It has been conducted annually since 2016. The survey data consisted of 127 items in six domains, namely, socio-demographic characteristics, employment, health status, living in Korea, Korean proficiency, and stay-related matters. In this study, 15 variables corresponding to self-rated health, individual factors, social factors, and living and working environment factors were selected in line with the study's purpose.

### Sample

**Figure 1:**
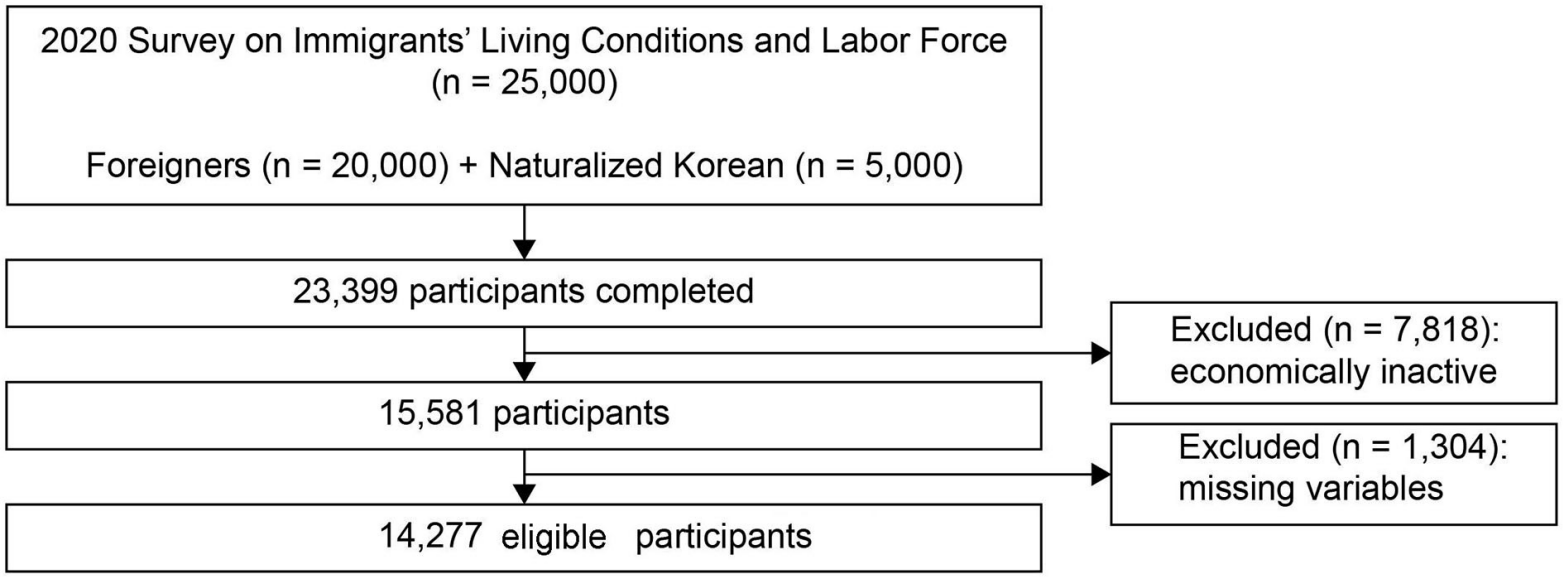
Flowchart of study participants' selection process.

The participants in this study were working or economically active immigrant workers. The survey sample comprised working-age foreigners aged 15 years and above who had been living in Korea for 91 days or longer (*N* = 20,000) and Korean citizens naturalized in the past 5 years (*N* = 5,000). The questionnaires comprised six parts: general characteristics, employment, health and informatization, living conditions, Korean language skills, and status of sojourn. The survey was conducted using two-phase sampling (the first phase was by district, and the second phase was based on visa status and nationality). It was conducted in participants' native languages by trained interviewers who visited participants' homes and workplaces. Of the administered questionnaires, 23,399 were completed. In this study, the economically inactive group (*n* = 7,818) and those who did not respond to the main study questions in the questionnaire (*n* = 1,304) were excluded. Therefore, the analysis was performed using data from 14,277 immigrant workers (Figure 1).

### Variables

#### Dependent variable: Self-rated health

Self-rated health is a simple and valid item to measure health status, and poor self-rated health is known to predict disease morbidity and mortality. The subjects responded to the question “How is your current overall health status?” by rating their health on a five-point Likert scale (very good, good, fair, poor, or very poor). Self-rated health has been used as a dichotomous variable in many previous studies and national comparisons of quality of life in health. Therefore, as in previous Korean national studies, this study dichotomized the results; “very good” and “good” were coded as 0, while “fair,” “poor,” and “very poor” were coded as 1 (16, 22). These response options were chosen from a previous study (15, 24) that revealed a preference for response options used to evaluate high-income countries. The Korean government conducts the survey on workers; immigrant workers positively evaluate their health status because they believe that a negative evaluation of their health status could be unfavorable for future employment.

#### Independent variables: Individual, social, and living and working factors

Following the literature, individual factors (gender, age, education, and ethnicity), social factors (length of residency, citizenship status, discrimination, Korean language ability, Korean natives they can ask for help, and unmet healthcare needs), and living and working environment factors (residential environment satisfaction, job satisfaction, income satisfaction, and work hours) were included as independent variables. Ethnicity was categorized as Korean–Chinese, Chinese, other Asian (East Asia, Southeast Asia), and non-Asian (America, Europe) based on the workers' nationality. Length of residency in Korea was categorized as <3 years, 3–9 years, and ≥10 years. Korean language ability was measured on a five-point Likert scale (1 = not proficient at all, 5 = very proficient) from the perspectives of speaking, listening, reading, and writing. Based on this information, the total score was calculated (total range of 4–20), and the participants were categorized into “fluent (16–20),” “moderately proficient (12–15),” and “limited (4–11).” Whether participants had access to Korean natives (colleagues or neighbors) whom they could ask for help in difficult situations was categorized into “yes” and “no.” Unmet healthcare needs refer to individuals being unable to fulfill their medical needs in the past year due to socioeconomic reasons, a lack of time, and/or language barriers. Subjective unmet healthcare needs and discrimination were added as binary variables. Working hours were categorized into three groups: temporary leave, <40 h/week, and ≥40 hours/week. Satisfaction with income, job satisfaction, and residential environment satisfaction were assessed using a five-point scale and then summed. The responses were categorized into “satisfied” (4 or 5 points), “moderately satisfied” (3 points), and “dissatisfied” (1 or 2 points).

### Ethical considerations

The original data are publicly available free of charge from the MicroData Integrated Service website (https://mdis.kostat.go.kr/index.do) for academic research. This study used a de-identified secondary dataset. Therefore, it was exempt from review by the Institutional Review Board of Daegu University, South Korea (no. 2021-041-08).

### Data analysis

Since data were obtained through stratified random sampling, all statistical analyses were conducted considering cross-sectional weights using SAS (version 9.4; SAS Institute, Cary, North Carolina, USA). First, descriptive analysis was conducted using frequencies and percentages for categorical variables and means and standard deviations for continuous variables. A Chi-square test and an independent samples *t*-test were performed to determine differences based on gender (Appendix 1). Second, to identify the factors associated with SRH, a multivariate survey logistic regression was conducted with associated factors for statistical adjustment. Subsequently, the odds ratios (ORs) and *p*-values (two-sided significance tests at 0.05) were calculated. Third, a series of multiple logistic regressions was conducted to explore factors that affected the SRH of immigrants. Model 1 was the simplest model. Model 2 was the model fully adjusted for the interaction of gender with the variables, including the following four factors: demographic factor (education level), social factor (length of residency), and living and working factors (residential environment satisfaction, work hours). Finally, interaction effect plots based on estimated predicted scores as marginal effects were presented using STATA 16.0 (Stata Corp, College Station, TX).

## Results

### Participants' characteristics

Table 1 presents the participants' general characteristics. Participants' descriptive statistics have been presented after dividing them into an unweighted sample (*N* = 14,277) and a weighted sample (*N* = 871,264). The overall percentage of poor SRH among participants was 23.0%.

**Table 1:** Baseline characteristics of the study sample.

**Variables**	**Unweighted *n***	**Weighted *n* (weighted %)**
	**(*n* = 14,277)**	**(*n* = 871,264)**
Self-rated health		
Good	10,779	670,862 (77.00)
Poor	3,498	200,402 (23.00)
*Individual factors*		
Gender		
Men	8,721	578,600 (66.41)
Women	5,556	292,644 (33.59)
Age (years)		
15–29	3,644	218,795 (25.11)
30–39	4,936	291,320 (33.44)
40–49	2,503	153,558 (17.62)
50–59	2,090	140,109 (16.08)
≥ 60	1,104	67,482 (7.75)
Education level		
≤ Elementary school	1,179	71,326 (8.19)
Middle school	2,797	164,001 (18.82)
High school	6,147	373,156 (42.83)
≥ College	4,154	262,780 (30.16)
Ethnicity		
Korean-Chinese	5,172	329,734 (37.85)
Chinese	1,021	46,317 (5.32)
Other Asian	6,880	411,809 (47.27)
Non-Asian	1,204	83,404 (9.57)
*Social factors*		
Length of residency, years		
< 3	3,196	221,077 (25.37)
3–9	6,843	426,630 (48.97)
≥10	4,238	223,557 (25.66)
Korean citizenship (visa status)		
Acquired	2,892	28,146 (3.23)
Not acquired	11,385	843,118 (96.77)
Discrimination experience		
Yes	2,991	178,011 (20.43)
No	11,286	693,253 (79.57)
Korean language ability (mean ± SD)	9.02 ± 3.05	
Fluent	2,212	149,414 (17.15)
Moderately fluent	5,450	337,040 (38.68)
Limited	6,615	384,809 (44.17)
Koreans they can ask for help		
Yes	6,137	331,353 (38.03)
No	8,140	539,911 (61.97)
Unmet healthcare needs		
Yes	762	50,403 (5.79)
No	13,515	820,861 (94.21)
*Living and working environment factors*		
Residential environment satisfaction		
Dissatisfied	298	19,878 (2.28)
Mid	2,291	141,455 (16.24)
Satisfied	11,688	709,931 (81.48)
Job satisfaction		
Dissatisfied	752	42,787 (4.91)
Mid	3,698	215,411 (24.72)
Satisfied	9,827	613,066 (70.37)
Income satisfaction		
Dissatisfied	1,781	100,943 (11.59)
Mid	4,430	258,241 (29.64)
Satisfied	8,066	512,080 (58.77)
Working hours (hours/week)		
Temporary leave	1,055	57,446 (6.59)
< 40	1,912	107,376 (12.32)
≥ 40	11,310	706,442 (81.08)

SD, Standard deviation; Mid, middle.

Regarding individual factors, approximately two-thirds of the participants were male (66.41%). Further, 33.44% were aged between 30 and 39 years, while 25.11% were aged between 15 and 29 years. Less than half of all participants were other Asians (47.27%), while 37.85% were Korean–Chinese.

Regarding social factors, half of the participants' length of residency (48.97%) was 3–9 years, and only 3.23% had Korean citizenship. Moreover, 20.43% had experienced discrimination. The Korean proficiency score was 9.02 ±3.05 on average (range 4–20), and 44.17% responded that they had limited Korean language ability. Additionally, 30% of participants had Korean colleagues or neighbors they could ask for help, and only 5.79% had experienced subjective unmet healthcare needs.

Regarding living and working environment factors, the majority of the participants were satisfied with their residential environment (81.48%), job (70.37%), and income (58.77%). Participants working over 40 h per week comprised 81.08% of the total participants.

All variables showed statistically significant differences according to gender (See Appendix 1). The proportion of men under the age of 39 years (62.90%) was higher than that of women (49.95%). Women reported more discrimination (23.88%) than men (18.69%) did. Most men (49.62%) and women (47.67%) had been in Korea for 3–9 years; however, the proportion of women (18.26%) living in Korea for < 3 years was lower than that of men (28.97%). The proportion of men (86.24%) who worked over 40 h per week was more than that of women. Further, the proportion of women who worked 20–39 h per week (18.60%) or were on temporary leave (10.52%) was more than that of men. The score for Korean language ability was higher for women than for men (9.66 vs. 8.70, *p* < 0.001).

### Factors affecting the self-rated health of immigrant workers

In Table 2, Model 1 presents the adjusted multivariate logistic regression results for factors affecting SRH. Individual, social, and living and working environment factors were significantly associated with poor SRH. Regarding individual factors, poor SRH increased with increasing age in this study, and older groups were more likely to report poor SRH. The higher education group showed significantly lower instances of poor SRH than the elementary school group. Chinese (OR = 0.73, 95% confidence interval [CI]: 0.71–0.76) and non-Asian workers (OR = 0.77, 95% CI: 0.756–0.79) were likely to have lower odds of reporting poor SRH than Korean–Chinese immigrants.

**Table 2:** Factors associated with the self-rated health of migrant workers with interactions.

**Variables**	**Model 1**			**Model 2**		
	**Adjusted OR**	**95% CI**	***p***	**Adjusted OR**	**95% CI**	***p***
Gender (ref: male)						
Female	1.48	1.47–1.50	< 0.001	1.89	1.78–2.01	< 0.001
Age (ref: 15–29)						
30–39	1.17	1.15–1.19	< 0.001	1.16	1.14–1.18	< 0.001
40–49	1.63	1.59–1.66	< 0.001	1.61	1.58–1.64	< 0.001
50–59	2.72	2.66–2.78	< 0.001	2.69	2.64–2.75	< 0.001
≥ 60	3.67	3.58–3.77	< 0.001	3.63	3.54–3.73	< 0.001
Education Level (ref: < Elementary school)						
Middle school	0.97	0.95–0.99	0.008	1.09	1.06–1.12	< 0.001
High school	0.95	0.93–0.97	< 0.001	1.06	1.03–1.09	< 0.001
≥ College	0.92	0.90–0.94	< 0.001	1.12	1.09–1.16	< 0.001
Ethnicity (ref: Korean-Chinese)						
Chinese	0.73	0.71–0.76	< 0.001	0.73	0.71–0.75	< 0.001
Other Asian	1.05	1.03–1.07	< 0.001	1.03	1.01–1.05	.008
Non-Asian	0.77	0.75–0.79	< 0.001	0.78	0.76–0.80	< 0.001
Residence length, years (ref: < 3)						
3–9	1.32	1.30–1.34	< 0.001	1.29	1.26–1.31	< 0.001
≥ 10	1.71	1.68–1.75	< 0.001	1.60	1.56-1.64	< 0.001
Korean citizenship (ref: Not acquired)						
Acquired	1.20	1.16–1.24	< 0.001	1.19	1.15–1.23	< 0.001
Discrimination experience (ref: No)						
Yes	1.26	1.24–1.28	< 0.001	1.26	1.25–1.28	< 0.001
Korean language ability	0.97	0.97–0.98	< 0.001	0.97	0.97–0.98	< 0.001
Koreans who they can ask for help (ref: Yes)						
No	1.11	1.09–1.12	< 0.001	1.11	1.09–1.12	< 0.001
Unmet healthcare needs (ref: No)						
Yes	3.07	3.00–3.13	< 0.001	3.07	3.01–3.14	< 0.001
Residential environment satisfaction (ref: Satisfied)						
Mid	1.49	1.47–1.51	< 0.001	1.46	1.43–1.48	< 0.001
Unsatisfied	1.80	1.74–1.86	< 0.001	1.04	1.00–1.09	0.077
Job satisfaction (ref: Satisfied)						
Mid	2.23	2.20–2.26	< 0.001	2.24	2.20–2.27	< 0.001
Unsatisfied	2.07	2.02–2.13	< 0.001	2.08	2.02–2.13	< 0.001
Income satisfaction (ref: Satisfied)						
Mid	1.47	1.45–1.49	< 0.001	1.48	1.46–1.50	< 0.001
Unsatisfied	1.40	1.37–1.42	< 0.001	1.39	1.36–1.42	< 0.001
Work hours (ref: Temporary leave)						
< 40	0.73	0.71–0.74	< 0.001	0.74	0.71–0.76	< 0.001
≥40	0.81	0.79–0.83	< 0.001	0.83	0.80–0.86	< 0.001
Gender × education (ref: men × < Elementary school)						
Women × Middle school				0.77	0.74–0.81	< 0.001
Women × High school				0.77	0.74–0.80	< 0.001
Women × ≥ College				0.61	0.58–0.63	< 0.001
Gender × length of residency (ref: men × < 3 years)						
Women × 3–10 years				1.07	1.03–1.10	< 0.001
Women × ≥ 10 years				1.14	1.10–1.18	< 0.001
Gender × Residential environment satisfaction (ref: men × Satisfied)						
Women × Moderate				1.06	1.03–1.09	0.001
Women × Unsatisfied				3.61	3.37–3.87	< 0.001
Gender × Working hours (ref: men × Temporary leave)						
Women × < 40 h				0.96	0.91–1.01	0.089
Women × ≥ 40 h				0.92	0.88–0.96	< 0.001
Model fit						
AIC	768292.28				766278.96	
SC	768494.73				766548.89	
2 Log L	768238.28				766206.96	

OR, Odds ratio; CI, confidence interval.

Regarding social factors, participants had a higher likelihood of reporting poor SRH when their length of residency was < 3 years (3–9 years OR = 1.32, 95% CI: 1.30–1.34; over 10 years OR = 1.71, 95% CI: 1.68–1.75). Poor SRH increased when participants had Korean citizenship (OR = 1.20, 95% CI: 1.61–1.24) or had experienced discrimination (OR = 1.26, 95% CI: 1.24–1.28). Additionally, individuals who did not have social support from colleagues or neighbors (i.e., Korean natives) showed a higher likelihood of poor SRH (OR = 1.11, 95% CI: 1.09–1.12). Fluency in Korean was negatively associated with poor SRH (OR = 0.97, 95% CI: 0.97–0.98). Participants with unmet healthcare needs (OR = 3.07, 95% CI: 3.00–3.17) were more likely to report poor SRH than their counterparts.

Regarding living and working environment factors, participants who answered that they were less satisfied with their residential environment (mid OR = 1.49, 95% CI: 1.47–1.51; unsatisfied OR = 1.80, 95% CI: 1.74–1.86), job (mid OR = 2.23, 95% CI: 2.20–2.26; unsatisfied OR = 2.07, 95% CI: 2.02–2.13), and income (mid OR = 1.47, 95% CI: 1.45–1.49; unsatisfied OR = 1.40, 95% CI: 1.37–1.42) were more likely to report poor SRH than those who were satisfied.

In Model 2, multivariate logistic models (with or without interactions) were estimated and measured to identify interaction effects (gender × education, length of residency, residential environment satisfaction, and working hours per week).

The independent variables of Model 2, which had statistically significant results, were identical to those of Model 1, with similar ORs. These included gender, individual, social, and living and working environment factors, all of which significantly affected poor SRH. Significant interactions of gender and other variables were found: gender × education; gender × length of residency; gender × residential environment satisfaction; and gender × working hours per week (female × ≥40 h, *p* < 0.001; female < 40 h, *p* = 0.089).

**Figure 2:**
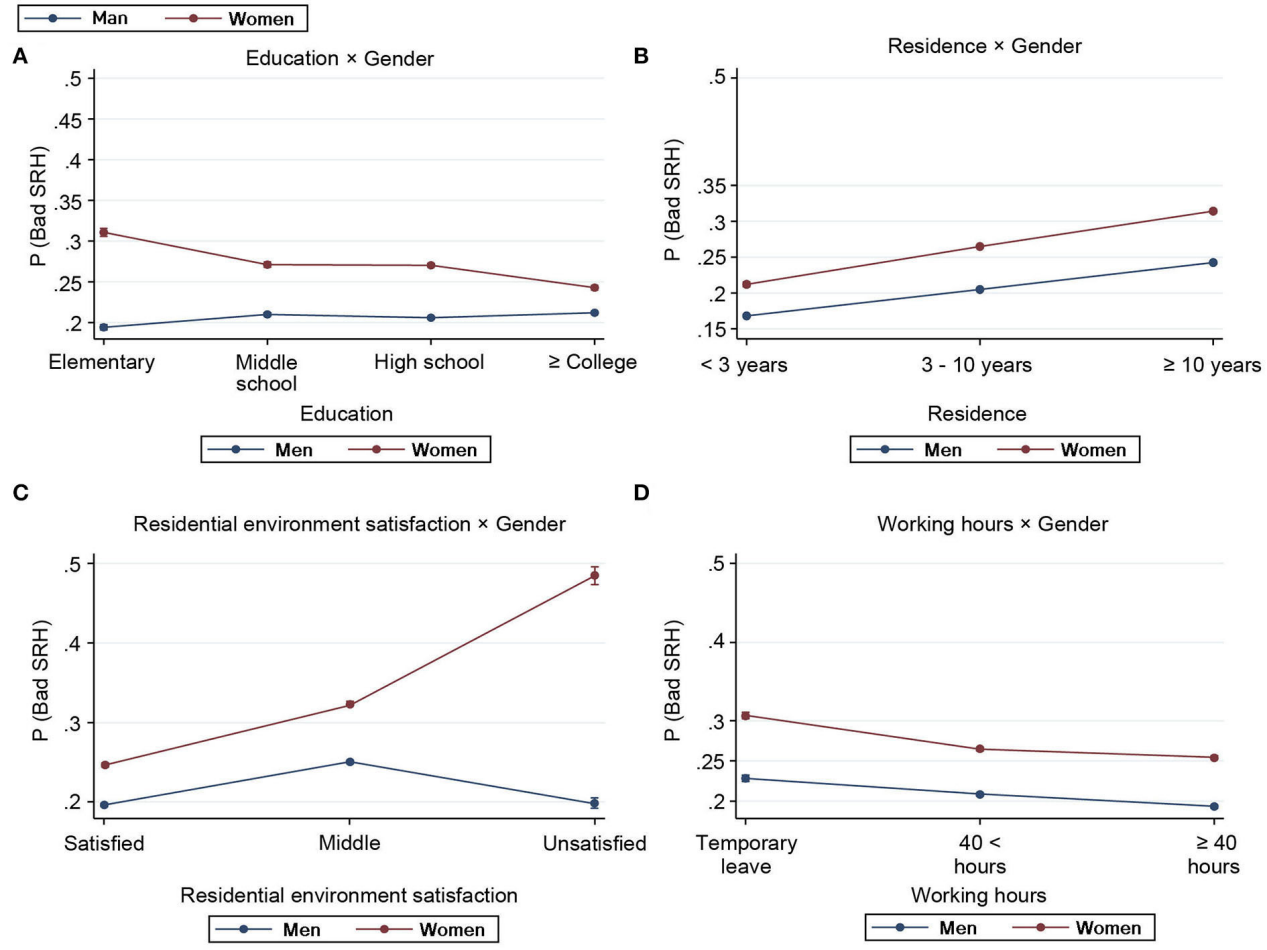
Estimated scores and marginal effects of the interaction effects of gender with the variables of education **(A)**, length of residence **(B)**, residential environment satisfaction **(C)**, and working hours **(D)**.

Model 2 demonstrated that women's poor SRH decreased when their education level increased [female × college OR = 0.61 vs. female × middle OR = 0.77]. Figure 2 demonstrates the estimated scores and marginal effects of the interaction effects of gender with the aforementioned variables. In Figure 2A, the impact of the interactions between gender and level of education on SRH revealed that men's poor SRH estimation did not improve when their level of education progressed from below elementary school to college and above. By contrast, women demonstrated a decreasing probability of poor SRH estimation as their level of education increased. This was interpreted as an improvement in their subjective health status. The interaction between gender and length of residency demonstrated a higher likelihood of poor SRH in women as their length of residency increased from 3 to 9 years to above 10 years [female × 3–10 years OR = 1.07 vs. female × above 10 years OR = 1.14]. Figure 2B demonstrates the difference in the probability of poor SRH according to the length of residence between men and women. The OR of poor SRH was 3.61; it was the highest when women were unsatisfied with their residential environments. Figure 2C demonstrates that women who were unsatisfied with their residential environment were more likely to exhibit poor SRH than men who were satisfied with their residential environment. Regarding the interaction effect between gender and working hours, SRH was worse for those on temporary leave than for those with higher working hours.

## Discussion

This study investigated the self-rated health status and its influencing factors among working immigrants in South Korea. The results of this study should be insightful to researchers interested in studying immigrants' health and to immigration policy developers. Previous related studies were limited to a specific group such as married immigrant women and Korean–Chinese workers in Korea. This study considered a diverse immigration population compared to previous studies. It comprehensively investigated the relationship between individual, social, living and working environment factors, and immigrant workers' self-rated health using a nationally representative large sample in Korea. Therefore, the results are meaningful in that they can provide more comprehensive information to researchers and governments from the Korean perspective. In total, 23% of the participants rated their health as poor. This finding is similar to the results (24.1%) of a previous survey conducted in 2018 (15), and it did not demonstrate a considerable difference from the poor SRH (23.3%) of native Korean wage workers in a previous survey (*n* = 21,476) (25). Numerous studies have reported that migrant workers have lower health statuses than the native population (26). The results of this study on immigrant workers in Korea demonstrated better SRH than the study results (27.3–30.7%) for 14 Western European countries (11). However, some considerations must not be overlooked. First, social desirability, whereby immigrant workers in Korea rate their SRH more positively than it is in reality, may have influenced immigrants' SRH. This survey was conducted by the Korean government, and workers may have evaluated their health affirmatively because they believed that negative evaluations of their health status would place them at a disadvantage in their future employment (15). Second, the response format for assessing SRH should also be considered. While studies in the United States and Canada used an asymmetrical scale (excellent, very good, good, fair, and poor), other OECD countries have used symmetrical responses (very good, good, fair, poor, and very poor). In this study, symmetrical responses were used. Therefore, researchers must interpret these findings while considering possible comparative bias (27).

Further, this study also identified various factors that might affect the SRH of immigrants at the individual, social, and environmental levels. Regarding individual factors, older workers were found to be more likely to report poor SRH. Moreover, educational level and age exerted significant effects on SRH for women rather than for men. SRH improved among women who had at least college-level education. To understand these differences, the characteristics of the immigrant population must be investigated. Over 85% of this study's sample comprised Korean–Chinese (37.86%) workers or participants from other Asian countries (47.27%). Korean–Chinese people, who are of Korean descent but hold Chinese citizenship, are the largest ethnic group of immigrant workers in South Korea. The majority of them are employed as restaurant workers or domestic helpers, and among them, approximately 60% are middle-aged women (28). By contrast, a higher number of non-professional immigrant workers from Southeast or Southwest Asian countries are employed in the manufacturing, agriculture, and construction sectors through the Employment Permit System (EPS). A majority of them (approximately 90%) are male workers (17). However, although both professional and non-professional workers are low-wage workers in labor-intensive sectors, their educational qualifications and socioeconomic characteristics differ. Many non-professional immigrant workers are engaged in low-wage jobs regardless of their educational level. According to a recent report (17), Korean–Chinese migrants have lower education levels than non-professional migrant workers. Therefore, it must be considered that the education level of non-skilled immigrant male workers does not help them secure a job in Korea. The inverse relationship between educational level and the SRH of immigrant workers may be partly explained by men having a low socioeconomic status compared to their educational level as they work in low-skill and low-wage jobs.

Considering social factors, immigrants with longer lengths of residency in Korea, citizenship status, perceived discrimination, no social support from colleagues and neighbors, and unmet healthcare needs were more likely to report poor SRH than their counterparts. In this study, immigrant workers who had been in Korea for over 3 years reported worse SRH statuses. This is consistent with the disappearance of healthy immigrant effects over time, a phenomenon in which newly arrived immigrants are healthier than comparable native populations (8, 29). Citizenship status, discrimination experience, social relationships with natives, and access to medical services are social determinants of immigrants' health statuses (30, 31). In this study, only 3.23% of the workers had acquired Korean citizenship, and 77.5% of them were women. According to the statistical data (2), Korean–Chinese individuals constitute the highest proportion of immigrants with Korean citizenship, followed by Vietnamese and Filipino immigrants. It is not easy for foreigners, such as those on a migrant worker visa, to meet the requirements for obtaining citizenship as it is based on a points-based system that considers the duration of residence, property ownership, income thresholds, and individuals' Korean language proficiency. Therefore, obtaining citizenship is a process that requires considerable effort and energy and might predispose immigrants to poor health. Discrimination experience was found to lead to a decline in SRH, which is consistent with the results of previous studies (9, 10, 30). Approximately 20% of immigrants experienced discrimination in Korea in this study, but Choi and Song (2018) (32) reported that 77.2% of migrant workers perceived discrimination in their life. Perceived discrimination increases psychological distress and negatively affects an individual's physical and mental health (33); therefore, the Korean government must monitor discrimination and develop strategies to reduce the discrimination experienced by migrant workers. In this study, 61.97% of workers did not have native Koreans to assist and support them. Under the current EPS, the Korean Government does not allow migrant workers to settle with their families. The lack of support from their family is considered one of the leading causes of mental health problems in immigrant workers. Further, 44.17% of workers in this study had limited Korean language proficiency. Language proficiency makes it possible to adapt well to life in Korea, obtain a suitable job, and lead a stable life. According to prior studies, many migrant workers have language and communication difficulties in their lives (18), and these difficulties have been found to be one of the main barriers to using healthcare services (28). In this study, those with unmet healthcare needs reported higher odds ratios of poor SRH (OR = 3.07) than other factors. This result is consistent with a previous study's finding (OR = 3.63) (15). However, among the participants in this study, 5.79 % reported an unmet healthcare need over the past year. This is a lower prevalence than in previous studies, which reported that 17.90% of migrant workers (17) and 11.6% of working immigrant women (19) had unmet healthcare needs, based on a nationwide sample in Korea. In addition, this study found that women are 4.11 times more likely than men to have an unmet healthcare need. This result is also low compared to the figure of 9.5 %, revealed by a previous study on native Korean adults aged 19 and over (34). Since unmet healthcare needs are an important indicator of health disparity, the Korean government must monitor this issue and develop strategies to decrease the number of unmet healthcare needs.

Regarding living and working environment factors, poor residential environment, job, and income satisfaction, along with fewer working hours were associated with poor SRH. Residential satisfaction is one of the predisposing factors determining an individual's quality of life. Despite existing universal welfare policies, most immigrants and minority ethnic groups experienced disadvantages in their residential environment, including housing (35). Recently, the increased COVID-19 infection cases among Singapore's migrant workers who were living collectively in poor, densely populated spaces highlight the importance of safe residence environments and workplaces for vulnerable immigrants (36). In this study, the number of participants dissatisfied with their residential environments was small (*n* = 298); however, gender differences were observed among those dissatisfied with their residential environments. An interaction effect for gender was identified, and women who were dissatisfied with their residential environment were 3.87 times more likely to report poor SRH than men who were satisfied with their residential environment, suggesting that women are more affected by living conditions than men. This survey does not provide housing data in detail; however, according to a previous study (17), most male immigrants live in company dormitories (43.5%), while female immigrants are more likely to live in residences with a monthly rental agreement (75.0%). Considering the economic burden of housing costs (17), women prefer to stay in places with low monthly rents, which often exposes them to poor and unsafe living environments. Therefore, a safe housing policy must be urgently established, and residential satisfaction must be increased for female immigrant workers. Immigrant workers are known to work longer hours than native workers. Under the revised Labor Standards Act, the Korean government began to adopt a 40-h workweek in 2004. In this study, 81.08% of the participants worked over 40 h per week, and in a previous Korean study (17), 24.6% of immigrant workers worked over 50 h per week. Long working hours can adversely affect worker safety and health status (37). However, the results are not always consistent. In this study, those with a temporary leave status and those unsatisfied with their job and income were more likely to have poor SRH. Further, temporary leave status was small at 6.59%. However, as immigrants do not easily quit their jobs despite their dissatisfaction with their working conditions due to their visa status or economic vulnerability (17), attention should be given to the fact that their health status is poor while on temporary leave. Immigrants work in precarious conditions and are exposed to dangerous substances in the workplace (26). Furthermore, they have reported more health problems than native workers (38). The poor living and working environments of immigrant workers are becoming an issue in Korean society (39, 40). According to this study's findings, female workers are a vulnerable health population; therefore, to develop healthcare policies for immigrant workers, immigration policymakers and researchers should first consider female workers when developing healthcare policies that account for Korea's living and working environments.

## Conclusion

This study analyzed representative nationwide survey data of a large sample of immigrant workers in Korea. The study investigated the individual, social, and living and working environment factors that influence immigrant workers' SRH. Unmet health needs, low job satisfaction, low residential environment satisfaction, length of residence, and discrimination experience were found to worsen SRH. Therefore, to enhance the health status of immigrants, the Korean government should develop strategies to decrease immigrants' unmet healthcare needs. Furthermore, the work environment and related policies should consider immigrant workers' characteristics by identifying the socioeconomic characteristics that affect their health statuses.

### Limitations

This study has some limitations. First, due to the limited information provided in the original survey, the physical working environment, health behaviors, and emotional states of migrant workers, which could affect workers' health, could not be included. Second, although SRH is the most widely used indicator to monitor and summarize one's own health (5), whether participants' ratings of their health status are entirely reliable and accurate is not clear. In other words, the definition or standard of health in the social and cultural contexts to which the respondent belongs may have affected their SRH response (5). Migrant workers tend to portray their health status as being more positive than it really is; they may be influenced by social desirability because they are concerned about the potential disadvantages they may face in the labor market. Objective criteria must support the subjective health status assessments of immigrant workers. Therefore, to identify more empirical evidence on immigrant health status and influencing factors, future studies must use the Short Form-12 Health Survey Questionnaire (SF-12) (41) or objective health indicators. Third, since this study's data collection involved a self-report method, the participants' responses may have been biased, and the relationship between poor SRH level and its influencing factors may be overestimated or underestimated. Therefore, these aspects must be considered when interpreting or generalizing the study's results. Finally, this study employed a cross-sectional design; therefore, a causal relationship among the variables cannot be inferred. Further longitudinal research is needed to study immigrants' health status and health outcomes in Korea.

## Data availability statement

Publicly available datasets were analyzed in this study. This data can be found here: MicroData Integrated Service website (https://mdis.kostat.go.kr/mypage/extract/viewMyExtractListNew.do?curMenuNo = UI_POR_P9030), doi: 10.23333/P.920018.001.

## Ethics statement

The studies involving human participants were reviewed and approved by Institutional Review Board of Daegu University, South Korea. Written informed consent from the participants' legal guardian/next of kin was not required to participate in this study in accordance with the national legislation and the institutional requirements.

## Author contributions

SK contributed to the idea and design of this study. JH and DK undertook the data analysis and prepared the tables and figures. BK wrote the main manuscript text draft. SK and BK revised and finalized the manuscript. All authors read and approved the final manuscript.

## Funding

This study was supported by the Basic Science Research Program through the National Research Foundation of Korea (NRF) fund (Grant No. 2019R1I1A3A01062896).

## Conflict of interest

The authors declare that the research was conducted in the absence of any commercial or financial relationships that could be construed as a potential conflict of interest.

## Publisher's note

All claims expressed in this article are solely those of the authors and do not necessarily represent those of their affiliated organizations, or those of the publisher, the editors and the reviewers. Any product that may be evaluated in this article, or claim that may be made by its manufacturer, is not guaranteed or endorsed by the publisher.

## Abbreviations

OR: Odds ratios
SRH: Self-rated health
OECD: The Organization for Economic Co-operation and Development
SF-12: Short Form-12 Health Survey Questionnaire.
