# Supplementary material for: Factors associated with self-rated health among immigrant workers in South Korea: Analyzing the results of the 2020 survey on immigrants' living conditions and labor force
Source: Front Public Health. 2022 Sep 23;10:933724. doi: 10.3389/fpubh.2022.933724 (PMC9539430; doi:10.3389/fpubh.2022.933724)
Supplement: Supplementary file 1 [file Data_Sheet_1.docx]

Supplementary Material

# Supplementary Tables

**Appendix 1. Baseline characteristics of the study population by gender**

| Variables | Men | | | | Women | | | | | Rao-Scott or t | | p | |  |
| --- | --- | --- | --- | --- | --- | --- | --- | --- | --- | --- | --- | --- | --- | --- |
|  | Unweighted n | | Weighted n (weighted %) | | | | Unweighted n | Weighted n (weighted %) | |  |  |  |  |  |
|  | N=8,721 | | N=578,600 | | N=5,556 | | | N=292,664 | |  |  |  |  |  |
| Self-rated health | | | | | | | | | | | | | |  |
| Good | 6,926 | | 466,468 (80.62) | | 3,853 | | | 204,394 (69.84) | | 12754.81 | | <.001 | |  |
| Poor | 1,795 | | 112,132 (19.38) | | 1,703 | | | 88,270 (30.16) | |  |  |  |  |  |
| *Individual factors* | | | | | | | | | | | | | |  |
| Age, years | | | | | | | | | | | | | |  |
| 15−29 | 2,328 | | 153,035 (26.45) | | 1,316 | | | 65,760 (22.47) | | 18910.47 | | <.001 | |  |
| 30−39 | 3,058 | | 210,882 (36.45) | | 1,878 | | | 80,438 (27.48) | |  |  |  |  |  |
| 40−49 | 1,523 | | 101,094 (17.47) | | 980 | | | 52,464 (17.93) | |  |  |  |  |  |
| 50−59 | 1,157 | | 75,001 (12.96) | | 933 | | | 65,108 (22.25) | |  |  |  |  |  |
| ≥ 60 | 655 | | 38,588 (6.67) | | 449 | | | 28,894 (9.87) | |  |  |  |  |  |
| Education level | | | | | | | | | | | | | |  |
| ≤Elementary | 633 | | 43,907 (7.59) | | 546 | | | 27,419 (9.37) | | 1348.06 | | <.001 | |  |
| Middle | 1,611 | | 107,439 (18.57) | | 1,186 | | | 56,563 (19.33) | |  |  |  |  |  |
| High | 3,888 | | 254,247 (43.94) | | 2,259 | | | 118,910 (40.63) | |  |  |  |  |  |
| ≥ College | 2,589 | | 173,008 (29.90) | | 1,565 | | | 89,772 (30.67) | |  |  |  |  |  |
| Ethnicity | | | | | | | | | | | | | |  |
| Korean-Chinese | 3,190 | | 197,263 (34.09) | | 1,982 | | | 132,471 (45.26) | | 32806.11 | | <.001 | |  |
| Chinese | 431 | | 20,573 (3.56) | | 590 | | | 25,744 (8.80) | |  |  |  |  |  |
| Other Asian | 4,391 | | 310,519 (53.67) | | 2,489 | | | 101,289 (34.61) | |  |  |  |  |  |
| Non-Asian | 709 | | 50,245 (8.68) | | 495 | | | 33,159 (11.33) | |  |  |  |  |  |
| *Social factors* | | | | | | | | | | | | | |  |
| Length of residency, years | | | | | | | | | | | | | |  |
| <3 | 2,351 | | 167,630 (28.97) | | 845 | | | 53,447 (18.26) | | 21083.51 | | <.001 | |  |
| 3−9 | 4,123 | | 287,121 (49.62) | | 2,720 | | | 139,510 (47.67) | |  |  |  |  |  |
| ≥10 | 2,247 | | 123,850 (21.41) | | 1,991 | | | 99,707 (34.07) | |  |  |  |  |  |
| Korean citizenship (visa status) | | | | | | | | | | | | | |  |
| Acquired | 1,041 | | 6,324 (1.09) | | 1,851 | | | 21,822 (7.46) | | 25174.03 | | <.001 | |  |
| Not acquired | 7,680 | | 572,276 (98.91) | | 3,705 | | | 270,842 (92.54) | |  |  |  |  |  |
| Discrimination experience | | | | | | | | | | | | | |  |
| Yes | 1,621 | | 108,123 (18.69) | | 1,370 | | | 69,888 (23.88) | | 3224.02 | | <.001 | |  |
| No | 7,100 | | 470,477 (81.31) | | 4,186 | | | 222,776(76.12) | |  |  |  |  |  |
| Korean language ability (mean±SD) | | 8.70± 25.26 | |  | | 9.66± 20.56 | | |  | | –18.93 | | <.001 | |
| Koreans they can ask for help | | | | | | | | | | | | | |  |
| Yes | 3,227 | | 199,332 (34.45) | | 2,910 | | | 132,021 (45.11) | | 9370.29 | | <.001 | |  |
| No | 5,494 | | 379,268 (65.55) | | 2,646 | | | 160,643 (54.89) | |  |  |  |  |  |
| Unmet health needs | | | | | | | | | | | | | |  |
| Yes | 428 | | 30,685 (5.30) | | 334 | | | 19,718 (6.74) | | 733.48 | | <.001 | |  |
| No | 8,293 | | 547,915 (94.70) | | 5,222 | | | 272,945 (93.26) | |  |  |  |  |  |
| *Living and working environmental factors* | | | | | | | | | | | | | |  |
| Residential environment satisfaction | | | | | | | | | | | | | |  |
| Dissatisfied | 159 | | 11,737 (2.03) | | 139 | | | 8,142 (2.78) | | 614.42 | | <.001 | |  |
| Mid | 1,348 | | 92,409 (15.97) | | 943 | | | 49,046 (16.76) | |  |  |  |  |  |
| Satisfied | 7,214 | | 474,454 (82.00) | | 4,474 | | | 235,476 (80.46) | |  |  |  |  |  |
| Job satisfaction | | | | | | | | | | | | | |  |
| Dissatisfied | 410 | | 24,724 (4.27) | | 342 | | | 18,063 (6.17) | | 4474.73 | | <.001 | |  |
| Mid | 2,070 | | 133,729 (23.11) | | 1,628 | | | 81,682 (27.91) | |  |  |  |  |  |
| Satisfied | 6,241 | | 420,147 (72.61) | | 3,586 | | | 192,919 (65.92) | |  |  |  |  |  |
| Income satisfaction | | | | | | | | | | | | | |  |
| Dissatisfied | 932 | | 57,096 (9.87) | | 849 | | | 43,847 (14.98) | | 12329.68 | | <.001 | |  |
| Mid | 2,474 | | 158,032 (27.31) | | 1,956 | | | 100,209 (34.24) | |  |  |  |  |  |
| Satisfied | 5,315 | | 363,473 (62.82) | | 2,751 | | | 148,607 (50.78) | |  |  |  |  |  |
| Working hours, hours | | | | | | | | | | | | | |  |
| Temporary leave | 446 | | 26,664 (4.61) | | 609 | | | 30,783 (10.52) | | 30047.58 | | <.001 | |  |
| <40 | 840 | | 52,934 (9.15) | | 1,072 | | | 54,442 (18.60) | |  |  |  |  |  |
| ≥40 | 7,435 | | 499,003 (86.24) | | 3,875 | | | 207,439 (70.88) | |  |  |  |  |  |

Notes: SD, Standard deviation; Mid, Middle

**Appendix 2. Factors affecting self-rated health of men and women**

| Variables | Men | | | Women | | |
| --- | --- | --- | --- | --- | --- | --- |
|  | OR | 95% CI | p | OR | 95% CI | p |
| *Individual factors* | | | | | | |
| Age (ref: 15−29) | | | | | | |
| 30−39 | 1.11 | 1.08–1.13 | <.001 | 1.26 | 1.22–1.30 | <.001 |
| 40−49 | 1.44 | 1.41–1.48 | <.001 | 1.93 | 1.87–2.00 | <.001 |
| 50−59 | 2.16 | 2.10–2.22 | <.001 | 3.66 | 3.53–3.79 | <.001 |
| ≥ 60 | 3.31 | 3.20–3.42 | <.001 | 4.32 | 4.15–4.51 | <.001 |
| Education level (ref: < Elementary) | | | | | | |
| Middle | 1.06 | 1.03–1.10 | <.001 | 0.87 | 0.84–0.90 | <.001 |
| High school | 1.02 | 0.99–1.05 | .180 | 0.86 | 0.84–0.89 | <.001 |
| ≥ College | 1.07 | 1.04–1.11 | <.001 | 0.75 | 0.72–0.78 | <.001 |
| Ethnicity (ref: Korean-Chinese) | | | | | | |
| Chinese | 0.82 | 0.78–0.86 | <.0001 | 0.66 | 0.64–0.69 | <.001 |
| Other Asian | 1.03 | 1.00–1.05 | .026 | 1.02 | 0.99–1.05 | .187 |
| Non-Asian | 0.82 | 0.80–0.85 | <.001 | 0.69 | 0.66–0.72 | <.001 |
| *Social factors* | | | | | | |
| Residence length, years (ref: <3) | | | | | | |
| 3−9 | 1.30 | 1.28–1.33 | <.001 | 1.34 | 1.31–1.39 | <.001 |
| ≥ 10 | 1.67 | 1.63–1.71 | <.001 | 1.73 | 1.68–1.79 | <.001 |
| Korean citizenship (ref: Not acquired) | | | | | | |
| Acquired | 1.12 | 1.05–1.20 | .000 | 1.29 | 1.24–1.34 | <.001 |
| Discrimination experience (ref: No) | | | | | | |
| Yes | 1.24 | 1.22–1.26 | <.001 | 1.31 | 1.29–1.34 | <.001 |
| Korean language ability (continuous) | 0.98 | 0.98–0.98 | <.001 | 0.95 | 0.94–0.95 | <.001 |
| Koreans who they can ask for help (ref: Yes) | | | | | | |
| No | 1.14 | 1.12–1.16 | <.001 | 1.04 | 1.02–1.06 | 0.004 |
| Unmet health needs (ref: No) | | | | | | |
| Yes | 2.61 | 2.55–2.68 | <.001 | 4.11 | 3.96–4.25 | <.001 |
| *Living and working environmental factors* | | | | | | |
| Residential environment satisfaction (ref: Satisfied) | | | | | | |
| Mid | 1.39 | 1.37–1.42 | <.001 | 1.63 | 1.59–1.67 | <.001 |
| Unsatisfied | 1.03 | 0.99–1.08 | 0.149 | 3.87 | 3.67–4.08 | <.001 |
| Job satisfaction (ref: Satisfied) | | | | | | |
| Mid | 2.41 | 2.37–2.45 | <.001 | 2.01 | 1.97–2.06 | <.001 |
| Unsatisfied | 1.99 | 1.92–2.06 | <.001 | 2.32 | 2.22–2.41 | <.001 |
| Income satisfaction (ref: Satisfied) |  |  |  |  |  |  |
| Mid | 1.61 | 1.58–1.64 | <.001 | 1.29 | 1.26–1.32 | <.001 |
| Unsatisfied | 1.38 | 1.34–1.42 | <.001 | 1.36 | 1.32–1.41 | <.001 |
| Work hours (ref: Temporary leave) | | | | | | |
| <40 | 0.73 | 0.70–0.76 | <.001 | 0.69 | 0.67–0.72 | <.001 |
| ≥40 | 0.82 | 0.80–0.85 | <.001 | 0.73 | 0.71–0.76 | <.001 |

Notes: OR, Odds ratio; CI, Confidence interval; Mid, Middle

**
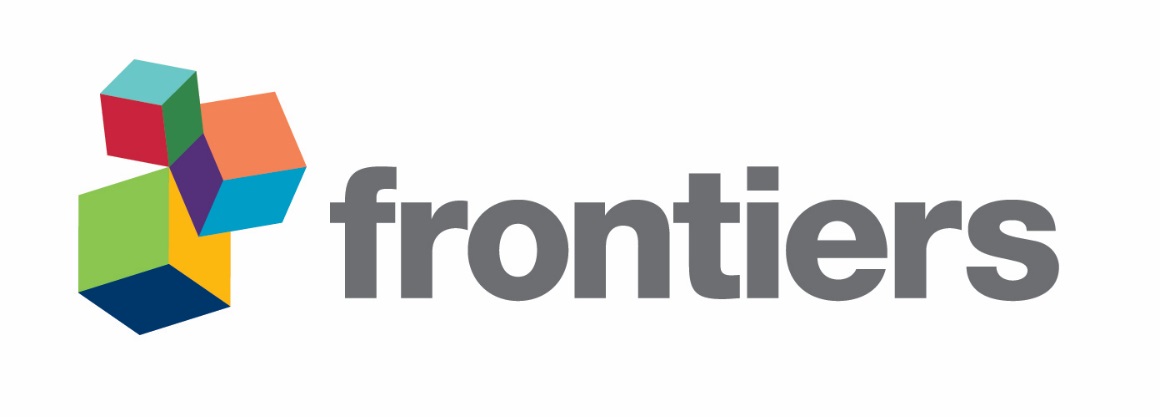
**
